# A R2R3-MYB Transcription Factor Gene, *BpMYB123*, Regulates *BpLEA14* to Improve Drought Tolerance in *Betula platyphylla*

**DOI:** 10.3389/fpls.2021.791390

**Published:** 2021-12-10

**Authors:** Kaiwen Lv, Hairong Wei, Guifeng Liu

**Affiliations:** ^1^State Key Laboratory of Tree Genetics and Breeding, Northeast Forestry University, Harbin, China; ^2^College of Forest Resources and Environmental Science, Michigan Technological University, Houghton, MI, United States

**Keywords:** abiotic stress, drought stress, *Betula platyphylla*, transcription factor, *BpMYB123*

## Abstract

Drought stress causes various negative impacts on plant growth and crop production. R2R3-MYB transcription factors (TFs) play crucial roles in the response to abiotic stress. However, their functions in *Betula platyphylla* haven’t been fully investigated. In this study, a R2R3 MYB transcription factor gene, *BpMYB123*, was identified from *Betula platyphylla* and reveals its significant role in drought stress. Overexpression of *BpMYB123* enhances tolerance to drought stress in contrast to repression of *BpMYB123* by RNA interference (RNAi) in transgenic experiment. The overexpression lines increased peroxidase (POD) and superoxide dismatase (SOD) activities, while decreased hydrogen peroxide (H_2_O_2_), superoxide radicals (O_2_^–^), electrolyte leakage (EL) and malondialdehyde (MDA) contents. Our study showed that overexpression of *BpMYB123* increased *BpLEA14* gene expression up to 20-fold due to BpMYB123 directly binding to the MYB1AT element of *BpLEA14* promoter. These results indicate that BpMYB123 acts as a regulator via regulating *BpLEA14* to improve drought tolerance in birch.

## Introduction

Drought is an adverse environmental condition as sessile plants have to cope with it at some point degrees in the their life cycle. When drought occurs, cells and tissues in plants are deprived of water, resulting in shrinkage, collapses, attenuated growth and eventually loss of biomass and productivity (Eziz et al., 2017). With the explosion of the world’s population and million metric tons of greenhouse gases, the global climate change increases the odds of worsening of drought in the world. In addition, drought alone causes more annual loss in crop yield than all other environmental factors combined (Ciais et al., 2005; Nadeem et al., 2019). Short-term drought has a direct destructive effect on the growth and production of annual crops (Wang et al., 2020) and can cause dieback of perennial woody plants. Long-term drought escalate the damage to forests, ecosystems, and wildlife (Gomes and Bernardino, 2020), and has the potential to alter forest composition and provoke the occurrence of tree-damaging forest insects and pathogens. Therefore, it is of great significance to understand the molecular mechanism of improving drought tolerance in plant (Zandalinas et al., 2018).

MYB transcription factor family plays an important role in the adaptive response to drought stress (Baldoni et al., 2015). The DNA-binding domains of MYB gene family consists of 51-52 amino acids, including three repeated sequences of R1, R2 and R3, each of which contains an H-T-H three-dimensional structure (Ogata et al., 1996). MYB is subdivided into four subfamilies according to the number of adjacent repeat units repeats: 4R-MYB, R1R2R3-MYB (3R-MYB), R2R3-MYB, and 1R-MYB (MYB-related) (Dubos et al., 2010).

In plants, the majority of MYB proteins belong to the R2R3-MYB subfamily that play central roles in response to drought stress, including root growth, stomatal movement, signaling transduction, homeostasis, and metabolite biosynthesis. For instance, *AtMYB96* plays a critical role in root growth under drought (Seo et al., 2009). *MdSIMYB*-overexpressing apple plant develops more robust root systems as compared to wild-type, and an improved tolerance to salt, drought and cold (Wang et al., 2014). Hypersensitivity to drought stress occurs to the transgenic overexpression plants of *AtMYB60*, a guard cell-specific gene (Oh et al., 2011), whereas the light-induced stomatal opening is repressed in the myb60 mutant, which enhances plant drought tolerance via less water loss (Cominelli et al., 2005). The silencing of *GbMYB5* gene decreases the drought tolerance in cotton seedlings, while the stomatal size and stomatal opening rate are significantly reduced in *GbMYB5* overexpression transgenic tobacco, resulting in the reduced the water loss and improved the survival rate under drought stress (Chen et al., 2015). The other example is *Arabidopsis thaliana AtMYB96*, whose overexpression transgenic lines exhibit normal growth and development and enhanced tolerance to drought. The deposition of epicuticular wax crystals increases significantly on the surfaces of transgenic leaves (Lee et al., 2014). *GmMYB14*, whose overexpression enhances the drought tolerance significantly through regulating plant architecture mediated by the brassinosteroid pathway. GmMYB14 binds to the promoter of *GmBEN1* and up-regulates its expression, resulting in decreasing the content of brassinosteroids (BRs) (Chen et al., 2021a). Furthermore, AtMYB12 regulates the key enzyme genes involved in the biosynthesis of flavonoid in the *AtMYB12-OE* transgenic lines, leading to the significant increase of the abscisic acid (ABA), proline, superoxide dismatase (SOD) and peroxidase (POD), and reduction of hydrogen peroxide (H_2_O_2_) and malondialdehyde (MDA) levels (Wang et al., 2016). In comparison with wild-type, the ABA content in *PtrMYB94* overexpression transgenic lines was significantly increased. *PtrMYB94* coordinates with ABA signaling and improves drought tolerance in *Populus* (Fang et al., 2019). Phytohormone signaling as key for regulating the response to drought (Gupta et al., 2020). In rice, overexpression of a R2R3-MYB gene, *SiMYB56*, from foxtail millet significantly enhances tolerance to drought stress whereas the lower MDA content and higher lignin and ABA contents were observed (Xu et al., 2020b).

Although MYB transcription factor has been studied extensively, R2R3-MYB gene functions in *Betula platyphylla* haven’t been fully investigated. In this study, we characterized the roles of R2R3-MYB transcription factor gene, *BpMYB123*, from *B. platyphylla* in response to drought stress. We found that the overexpression of *BpMYB123* in transgenic lines significantly improved the drought resistance of *B. platyphylla*, while down-regulation of *BpMYB123* using RNA interference (RNAi) decreased stress tolerance in repression transgenic lines. We used the yeast one-hybrid (Y1H) and chromosomal immunoprecipitation (ChIP) to show that BpMYB123 regulated the expression of *BpLEA14* by binding to the MYB1AT element in the promoter of *BpLEA14*, thereby improving the drought resistance of the plants. Overall, this study sheds new insights on drought tolerance mechanisms with broader implications for breeding drought tolerant varieties in *B. platyphylla*.

## Materials and Methods

### Vector Construction and Plant Transformation

We cloned the coding sequences (CDS) of *BpMYB123* whose gene identifier is BPChr03G03353 in recently published *B. platyphylla* genome (Chen et al., 2021b). Primers with adaptors containing specific restriction sites were designed for further cloning. Using cDNA of *B. platyphylla* as template, PCR products were cloned into PMD19-T vector for cDNA sequence validation. The CDS of *BpMYB123* was inserted into the binary vector pROK2 by using *Bam*HI and *Kpn*I double-enzyme digestion. Using the similar method, the CDS of *BpMYB123* (*Xba*I and *Sal*I) was cloned into binary vector of *pBI121-GFP*. The antisense fragments of *BpMYB123* were amplified by PCR, and then cloned into the binary vector *pROK2-RNAi*. The primer sequences used are shown in Supplementary Table 1.

The three binary vectors, *pROK2-BpMYB123*, *pBI121-BpMYB123-GFP* and *pROK2-RNAi-BpMYB123*, were transformed into *Agrobacterium* strain EHA105 by freeze-thaw method (Weigel and Glazebrook, 2006). The *B. platyphylla* transgenic lines were generated by the leaf disc method (Lv et al., 2020a). To identify true transgenic lines, DNA was extracted from transgenic plants using a DNA extraction kit (TIANGEN, Beijing, China) and was used for PCR. RNA was extracted from transgenic lines using the CTAB method (Chang et al., 1993) and used for qRT-PCR to calculate the expression levels of *BpMYB123* in transgenic lines. The primer sequences for PCR and qPCR are listed in Supplementary Table 1.

### RNA-Seq Data Analysis

Three *BpMYB123* overexpression lines were used for RNA-seq experiments. The RNA-Seq data was submitted to NCBI SRA database, and the BioProject ID was PRJNA776425. The clean reads were mapped to the *B. platyphylla* genome by using HISAT2 (Kim et al., 2019). The threshold of p-adjusted (padj) value <0.05 and fold change (FC) ≥2 were employed to identify the differentially expressed genes (DEGs). DEGs were annotated by eggNOG-mapper v2 (Cantalapiedra et al., 2021) and that were further analyzed by Gene Ontology (GO) enrichment analysis (Chen et al., 2020) to identify biological processes that were over-represented in DEGs.

### Transcription Factor-Centered Yeast One-Hybrid and Yeast One-Hybrid

The TF-centered Y1H was used to identify the binding *cis*-elements of BpMYB123 following the previously described method (Ji et al., 2014). Positive colonies were chosen and sequenced to identify candidate *cis*-element sequences.

To do Y1H, the CDS of *BpMYB123* was first cloned into the *pGADT7* vector (Clontech), while the promoter of *BpLEA14* was ligated into the pAbAi vector (Clontech). The construct, *pAbAi-BpLEA14*, was transformed into Y1HGold competent cells, and then cultured on SD/-Ura and SD/-Ura + AbA medium. Finally, *pGADT7-BpMYB123* plasmids were transformed into *pAbAi-BpLEA14* yeast competent cells, and cultured on SD/-Leu and SD/-Leu + AbA medium. The Y1H analysis was conducted using the Matchmaker Gold Yeast One-Hybrid Library Screening System Kit (Clontech). The primer sequences are listed in Supplementary Table 1.

### Chromosomal Immunoprecipitation Experiment and Chromosomal Immunoprecipitation-PCR

*pBI121-BpMYB123-GFP* transgenic plants were subjected to ChIP experiments following a method as described previously (Li et al., 2014) using anti-GFP antibodies. The precipitated DNAs were used as the templates for ChIP-PCR. There were six MYB1AT *cis*-elements in the promoter of *BpLEA14*, we designed the primers that span at the six sites. The primer sequences used are shown in Supplementary Table 1.

### Drought Stress Treatment

Birch seedlings were planted in soil in the greenhouse (16/8 h light/dark, 24°C) for two months. These seedlings were then used for drought treatment, the plants were placed in the greenhouse without watering for ten days. After that, the plants were irrigated for recovering. The phenotypes were the photographed.

To measure the physiological changes, the plants under drought treatment for seven days were harvested for measuring the activities of SOD and POD, the contents of H_2_O_2_, superoxide radicals (O_2_^–^), MDA and electrolyte leakage (EL). MDA, SOD and POD were measured following the methods as described previously (Wang et al., 2010), while EL was measured as described in our early publication (Lv et al., 2020b). H_2_O_2_ and O_2_^–^ were measured using the kit (Hydrogen peroxide content kit, H2O2-2-Y, Superoxide anion kit, SA-2-G, Suzhou Comin Biotechnology).

*B. platyphylla* seedlings were cultured in bottles pre-filled with 1/2 MS + 0.02 mg/L NAA + 2% (w/v) sucrose media, and then placed in a tissue culture room pre-set to 16/8-h light/dark cycles and an average temperature of 25°C for two months. They were then subjected to drought stress, and the leaves of these stressed plants were harvested for nitroblue tetrazolium (NBT) and 3, 3′-diaminobenzidine (DAB) stainings, as described previously (Zhang et al., 2011).

### Subcellular Localization of *BpMYB123*

In order to localize BpMYB123 proteins in the cells, the *pBI121-BpMYB123-GFP* plasmids were transferred into *Agrobacterium* strain EHA105, and then the agrobacteria were delivered into *Nicotiana benthamiana* epidermal cells using the injection method as described earlier (Pečenková et al., 2017). After the infection, the materials were placed at room temperature for 48 h. Fluorescence was observed and photographed under the confocal laser scanning microscope (LSM 800, Zeiss, Germany).

### Statistical Analysis

The student’s *t*-test was employed to test the statistically significant differences of various measures between *BpMYB123* transgenic lines and wild-type. The threshold for statistically significant differences was set to *p*-value <0.05.

## Results

### *BpMYB123* Gene Sequence and Subcellular Localization

**FIGURE 1:**
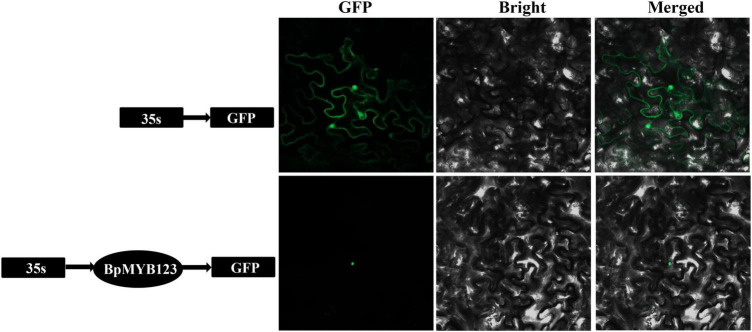
Subcellular localization analysis of BpMYB123.

Based on Sanger sequencing, CDS of *BpMYB123* is 915 base pairs (bp), which is 81 bp longer than 834bp of BPChr03G03353 from *Betula platyphylla* reference genome (Supplementary File 1). The subcellular localization experiment showed that BpMYB123 transcription factor was localized in the nucleus (Figure 1).

### Drought Tolerance of *BpMYB123* Transgenic Plants

**FIGURE 2:**
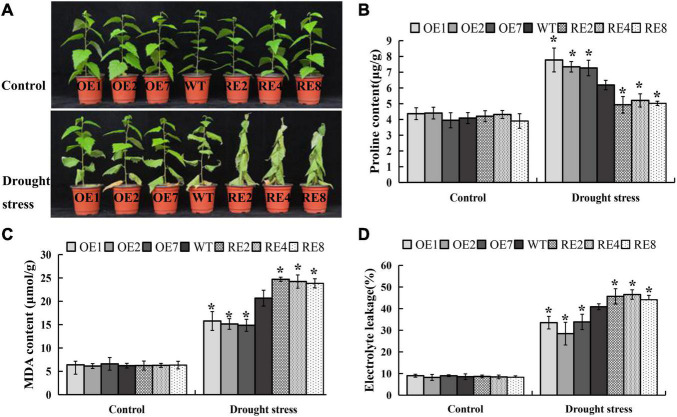
Overexpression of *BpMYB123* conferred enhanced drought tolerance in its transgenic lines. **(A)** Phenotypic comparison of *BpMYB123* transgenic lines and wild-type plants under drought stress. **(B)** Content of proline. **(C)** Content of malondialdehyde (MDA). **(D)** The result of electrolyte leakage. Asterisks indicate significant differences by *t*-test, *p* < 0.05.

Nine *BpMYB123* overexpression (*BpMYB123-OE*) transgenic lines, and eight *BpMYB123* repression (*BpMYB123-RE*) transgenic lines were obtained using leaf disc transformation method (Supplementary Figure 1). These transgenic lines and wild-type plants had been growing in the greenhouse for two months. After the drought treatment (without watering for 10-day) and a three-day watering recovery. Several leaves of *BpMYB123-OE* transgenic lines near the root shriveled and wilted, and half of them survived. In the wild-type, one of the third of the leaves survived, while all the leaves in the *BpMYB123-RE* transgenic lines withered (Figure 2A). We conclude that *BpMYB123* improved drought tolerance of *B. platyphylla*. We harvested the leaves after drought treatment for about a week and measured the contents of proline, MDA and EL (Figures 2B–D). The content of proline in *BpMYB123-OE* was significantly higher than wild-type (Figure 2B), whereas *BpMYB123-RE* transgenic lines were lower than wild-type. Plant cells subjected to stress can lead to membrane lipid peroxidation, damage of the structure of biofilm and changes in the permeability of cell plasma membrane. MDA is one of the final products of membrane lipid peroxidation. The integrity of the plasma membranes of the cells can be determined by the results of EL. The results of MDA content and EL in transgenic plants and wild-type cells before and after stress were shown in Figures 2C,D. Before drought stress, the content of MDA and EL in cells of all transgenic lines were largely the same without a significant difference. After drought stress treatment, the content of MDA and EL in cells increased, indicating that the cell membranes were oxidized. MDA was produced and the permeability of cell membrane was enhanced under drought stress. While the contents of MDA and EL in *BpMYB123-OE* transgenic lines were significantly decreased, the contents of MDA and EL in *BpMYB123-RE* transgenic lines were significantly increased in comparison with wild-type.

### Reactive Oxygen Species Scavenging in *BpMYB123* Transgenic Lines

**FIGURE 3:**
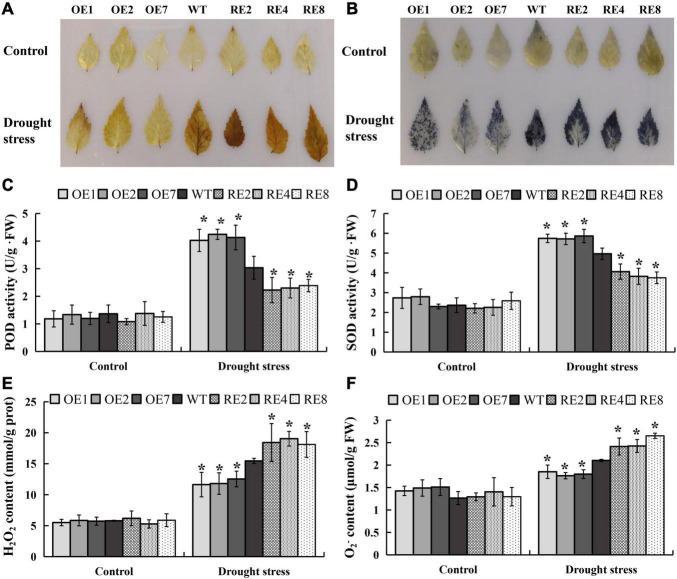
Reactive oxygen species (ROS) scavenging analyses in *BpMYB123*-overexpression (*BpMYB123-OE*) and *BpMYB123*-repression (*BpMYB123*-RE) transgenic lines and wild-type. **(A)** Content of hydrogen peroxide (H_2_O_2_) of *BpMYB123* different lines under drought stress by 3,3′-diaminobenzidine (DAB) staining. **(B)** Content of superoxide radicals (O_2_^–^) of *BpMYB123* different lines under drought stress by nitroblue tetrazolium (NBT) staining. **(C)** Content of peroxidase (POD). **(D)** Content of superoxide dismatase (SOD). **(E)** Content of H_2_O_2_. **(F)** Content of O_2_^–^. Asterisks indicate significant differences by *t*-test, *p* < 0.05.

Biochemical and physiological traits were measured to determine if the gain- and loss-of-function in *BpMYB123* transgenic lines under drought treatment. The DAB and NBT staining were used to measure H_2_O_2_ and O_2_^–^ levels; and thus represented the activities of POD and SOD. This is because POD and SOD are the enzymes responsive for removing H_2_O_2_ and O_2_^–^. After drought treatment, all three *BpMYB123-OE* lines showed significantly reduced H_2_O_2_ and O_2_^–^ levels compared to wild-type (Figures 3A,B). POD and SOD enzyme activities in the *BpMYB123-OE* transgenic lines were significantly higher than those in the wild-type (Figures 3C,D). Correspondingly, the contents of H_2_O_2_ and O_2_^–^ were significantly lower than those in the wild-type (Figures 3E,F). Thus, overexpression transgenic plants could eliminate reactive oxygen species (ROS) and diminish the toxicity of ROS.

### Identification and Validation of *BpMYB123*-Binding Motifs Using Yeast One-Hybrid

Using a TF-centered Y1H method, we obtained multiple yeast colonies containing pHIS2 element library plasmids with candidate sequence motifs, which could potentially be bound by BpMYB123 protein. We used the sequencing results to search the PLACE database^1^ and identify *cis*-elements in the inserted sequences. Some motifs were identified. There are six MYB1AT motifs in the promoter of *BpLEA14*. Because MYB1AT (WAACCA) is an element closely related to abiotic stress, and we considered it was the candidate motif (Table 1).

**TABLE 1:** *BpMYB123* bound *cis*-element.

Sequence	Core sequence	Motif prediction
ATTAACCAAT	WAACCA	MYB1AT

### RNA-Seq Data Analysis

1480 DEGs were identified in *BpMYB123-OE* lines as shown in Supplementary Table 2. Gene ontology (GO) enrichment analysis was performed using the DEGs form a pairwise comparison of *BpMYB123-OE* vs wild-type. The result showed a large number of DEGs responded to abiotic stress (Supplementary Table 3). There were 132 genes belonged to biological process of response to hormones (GO:0009725); 61 out of these 132 genes belong to biological process of respond to abscisic acid (GO:0009737), and many DEGs were responsive to metabolic process, such as cellular glucan metabolic process (GO:0006073); glycogen metabolic process (GO:0005977); energy reserve metabolic process (GO:0006112). According to the adjusted *p*-value, the top 20 GO terms were shown (Supplementary Figure 2).

BPChr06G29277 encodes a late embryogenesis abundant protein (LEA) and its protein sequence is highly homologous to AtLEA14 in *A. thaliana*; it was thus named *BpLEA14*. *BpLEA14* was significantly up-regulated (Log2 based fold change = 4.35, adjusted *p*-value = 6.04E-51) in *BpMYB123-OE* line*s*. We believe that *BpLEA14* plays an important role in enhancement of drought tolerance in *BpMYB123* transgenic plants.

### Identification of the Downstream Target Gene *BpLEA14* of *BpMYB123*

**FIGURE 4:**
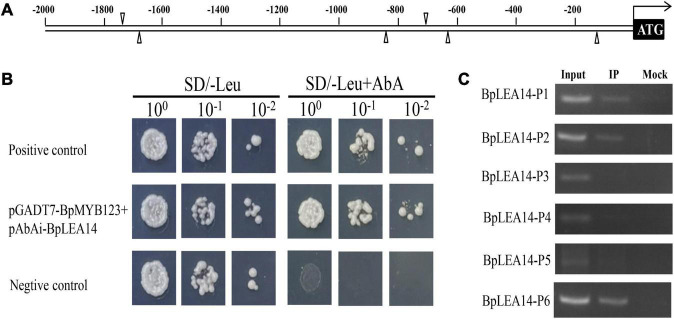
*BpLEA14* was the target gene of *BpMYB123*. **(A)** Proximal locations of *BpMYB123* binding *cis*-elements in *BpLEA14* promoter sequence. **(B)** BpMYB123 could bind to the promoter of *BpLEA14*. **(C)** Chromosomal immunoprecipitation (ChIP)-PCR analysis proved the binding of *BpMYB123* to *BpLEA14* promoter *in vivo* using an anti-GFP tag antibody for ChIP.

To confirm that BpMYB123 could bind to *BpLEA14*, we performed Y1H (Figure 4B). The result confirmed the binding of BpMYB123 to the promoter of *BpLEA14*.

ChIP-PCR was used to identify putative target gene of BpMYB123. There were six MYB1AT (WAACCA) *cis*-elements in the promoter of *BpLEA14* (Figure 4A). To verify that BpMYB123 could bind to *BpLEA14* by MYB1AT (WAACCA) motifs, we performed ChIP-PCR. After ChIP was done, the regions spanning the six MYB1AT motifs in *BpLEA14* promoter were amplified using PCR and the results were shown in Figure 4C. These results substantiated the binding of BpMYB123 to three MYB1AT (WAACCA) motifs in the promoter of *BpLEA14*.

### Drought Tolerance Assays of *BpLEA14* Overexpression Transgenic Lines

**FIGURE 5:**
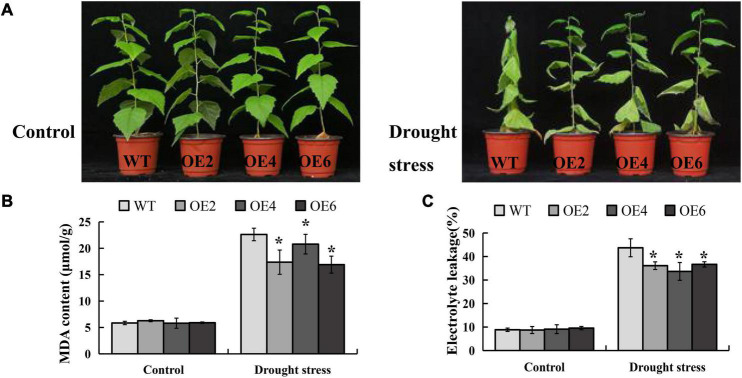
Overexpression of *BpLEA14* conferred enhanced drought tolerance in its transgenic lines. **(A)** Phenotypic comparison of *BpLEA14* overexpression (*BpLEA14-OE*) transgenic lines and wild-type (WT) plants under drought stress. **(B)** Content of malondialdehyde (MDA). **(C)** The result of electrolyte leakage. Asterisks indicate significant differences by *t*-test, *p* < 0.05.

The CDS of *BpLEA14* (456bp) was cloned from *B. platyphylla*, 1611bp shorter than the reference genomic region of BPChr06G29277 (2067bp). The sequence was shown in the Supplementary File 1. The CDS of *BpLEA14* was inserted into the binary vector pROK2. Seven transgenic lines were obtained by the leaf disc method, and three lines with the highest gene expression were selected for subsequent experiments (Supplementary Figure 3). Two-month-old *B. platyphylla* transgenic lines were grown in a greenhouse at 25°C under 16 h light/8 h dark period. Before the drought experiment was performed, all plants were fully irrigated. After 12 days without water, the plants were subjected to dehydration. The photos were taken three days later after the rehydration we initiated (Figure 5A). The content of MDA and EL in *BpLEA14* overexpression (*BpLEA14-OE*) transgenic lines were significantly decreased compared with wild-type (Figures 5B,C). That indicates *BpLEA14* improves drought tolerancein *B. platyphylla*.

## Discussion

MYB transcription factors play key regulatory roles to improve plant drought tolerance. For example, overexpression of an apple R2R3 gene, *MdoMYB121*, in tomato leads to lower MDA and EL levels and higher proline content in response to drought (Cao et al., 2013). In addition, overexpression of rice MYB-related type gene, *OsMYB48-1*, improves the tolerance to mannitol, PEG and NaCl in rice (Xiong et al., 2014), consequently, the transgenic lines show the improved tolerance to drought and the high salinity stress. Moreover, the activities of SOD, the contents of proline and of chlorophyll in tobacco transgenic lines overexpressing sugarcane R2R3-MYB subfamily gene, *SoMYB18*, increase significantly, resulting in the salt and drought stress tolerance of transgenic plants (Shingote et al., 2015). Finally, overexpression of *GmMYB118* in soybean improves tolerance to drought and salt stress through promoting expression of stress-associated genes and regulating osmotic and oxidizing substances to maintain cell homeostasis (Du et al., 2018).

In this study, we characterized the overexpression and repression transgenic lines of newly identified R2R3-MYB subfamily gene, *BpMYB123*, from *Betula platyphylla*. Under drought stress, the content of proline, activities of SOD and POD in *BpMYB123-OE* transgenic lines were significantly higher than those in wild-type, while the contents of H_2_O_2_ and O_2_^–^ were significantly lower. On the contrary, the *BpMYB123-RE* repression lines exhibited some opposite physiological responses and changes as compared to overexpression. Notably, the ability to remove reactive oxygen species of *BpMYB123-OE* lines were augmented under drought stress. These changes are largely aligned with the functions of some of above mentioned R2R3 MYB subfamily genes reported in other species.

Plant hormones and metabolites play an important role in stress response and tolerance in plants. Metabolic pathways could coordinately alleviate oxidative stress (Naderi et al., 2020), and hormone accumulation affects plant response to drought (Xu et al., 2017). In this study many DEGs associated with hormone and metabolic pathways (Supplementary Table 3). Gene ontology enrichment analysis suggests that *BpMYB123* regulates the hormone signaling and metabolic biosynthesis pathway under drought stress. This indicates that *BpMYB123* may modulate the levels of hormones and metabolites.

LEA protein is a large group of hydrophilic, glycine-rich protein members that exist in many plants (Amara et al., 2014), and play a key role in a variety of stresses (Gao and Lan, 2016). The accumulation of LEA protein has been reported to augment the tolerance of multiple stresses including cold (Xu et al., 2020a), salt (Aduse Poku et al., 2020) and drought (Yang et al., 2018). LEA genes play a determinant role in drought tolerance in rice (Kamarudin et al., 2019); for example, over-expression of *OsLEA3-1* in rice improves drought resistance (Xiao et al., 2007). In this study, over-expression of *BpMYB123* caused more than 20-fold increase of *BpLEA14*, and *BpLEA14* enhanced drought tolerance presumably by inhibiting cell membrane damage in *B. platyphylla* because over-expression of *BpLEA14* reduced the relative electrical conductivity, and malondialdehyde content under drought stress in *BpLEA14-OE* transgenic lines.

**FIGURE 6:**
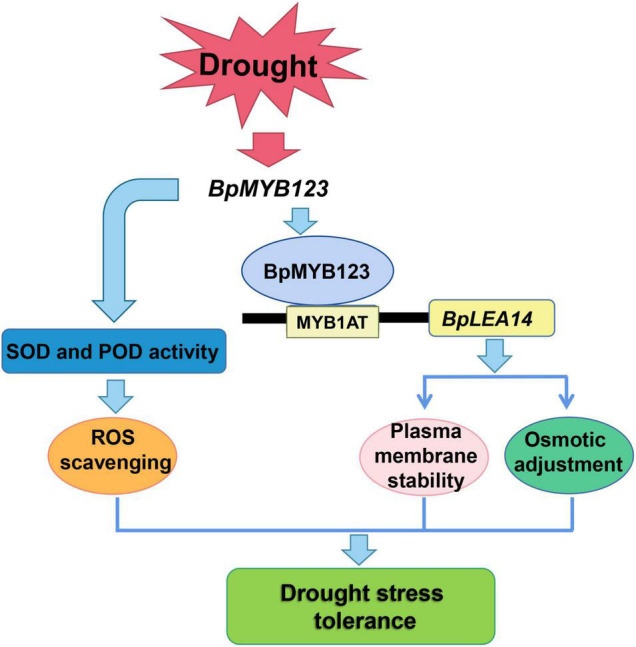
Working model of *BpMYB123* in response to drought stress.

Based on the above discussions and analyses, a schematic diagram sketching the molecular mechanism of how *BpMYB123* enhanced stress tolerance in *B. platyphylla* was drawn (Figure 6). Under drought stress, *BpMYB123* could enhance the ability of scavenging activity by boosting the activities of POD and SOD in transgenic plants. On the other hand, BpMYB123 bound to the MYB1AT *cis*-elements in the *BpLEA14* promoter to regulate the expression of *BpLEA14*, improving the stability of cell membrane and enhancing the drought resistance of the plants.

## Data Availability Statement

The original contributions presented in the study are publicly available. This data can be found here: https://www.ncbi.nlm.nih.gov/bioproject/, PRJNA776425.

## Author Contributions

KL and GL designed the study. KL performed most experiments. KL, HW, and GL wrote the manuscript. All authors contributed to the article and approved the submitted version.

## Conflict of Interest

The authors declare that the research was conducted in the absence of any commercial or financial relationships that could be construed as a potential conflict of interest.

## Publisher’s Note

All claims expressed in this article are solely those of the authors and do not necessarily represent those of their affiliated organizations, or those of the publisher, the editors and the reviewers. Any product that may be evaluated in this article, or claim that may be made by its manufacturer, is not guaranteed or endorsed by the publisher.

## Footnotes

1
https://www.dna.affrc.go.jp/PLACE/

## Supplementary Material

Supplementary Figure 1 Identification of *BpMYB123* transgenic lines. **(A)** The PCR identification of *BpMYB123-*overexpression (*BpMYB123-OE*) transgenic lines, M: DL2000 DNA Marker, N1: negative control (ddH_2_O), N2: negative control (wild-type of *Betula platyphylla*), P: positive plasmid, 1-9: different transgenic lines. **(B)** The qRT-PCR identification of *BpMYB123-OE* lines. **(C)** The PCR identification of *BpMYB123-*repression (*BpMYB123-RE*) lines. **(D)** The qRT-PCR identification of *BpMYB123-RE* lines. Asterisks indicate significant differences by *t*-test, *p* < 0.05.

Supplementary Figure 2 Gene ontology (GO) enrichment analysis. According to the adjusted *p*-value, the top 20 GO terms were shown.

Supplementary Figure 3 Identification of *BpLEA14* transgenic lines. **(A)** The PCR identification of overexpression transgenic plants of *BpMYB14*, M: DL2000 DNA Marker, N1: negative control (ddH_2_O), N2: negative control (wild-type of *Betula platyphylla*), P: positive plasmid, 1-7: different transgenic lines. **(B)** The qRT-PCR identification of *BpLEA14* overexpression (*BpLEA14-OE*) transgenic plants. Asterisks indicate significant differences by *t*-test, *p* < 0.05.
