# Supplementary figures and images for: A R2R3-MYB Transcription Factor Gene, BpMYB123, Regulates BpLEA14 to Improve Drought Tolerance in Betula platyphylla
Source: Front Plant Sci. 2021 Dec 10;12:791390. doi: 10.3389/fpls.2021.791390 (PMC8702527; doi:10.3389/fpls.2021.791390)

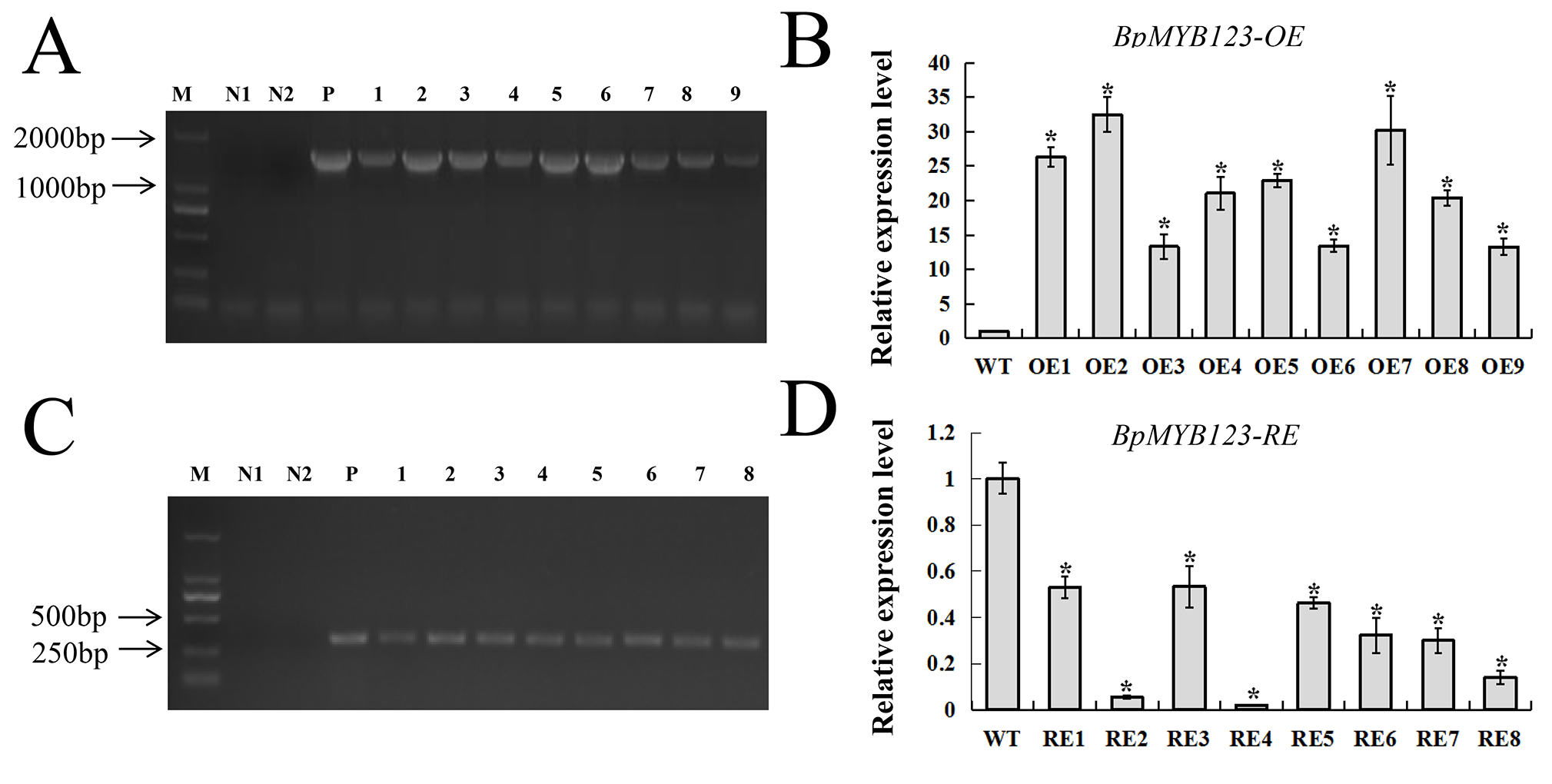

Supplement: Supplementary Figure 1 — Identification of BpMYB123 transgenic lines. (A) The PCR identification of BpMYB123-overexpression (BpMYB123-OE) transgenic lines, M: DL2000 DNA Marker, N1: negative control (ddH2O), N2: negative control (wild-type of Betula platyphylla), P: positive plasmid, 1-9: different transgenic lines. (B) The qRT-PCR identification of BpMYB123-OE lines. (C) The PCR identification of BpMYB123-repression (BpMYB123-RE) lines. (D) The qRT-PCR identification of BpMYB123-RE lines. Asterisks indicate significant differences by t-test, p < 0.05. [file Image_1.TIF]

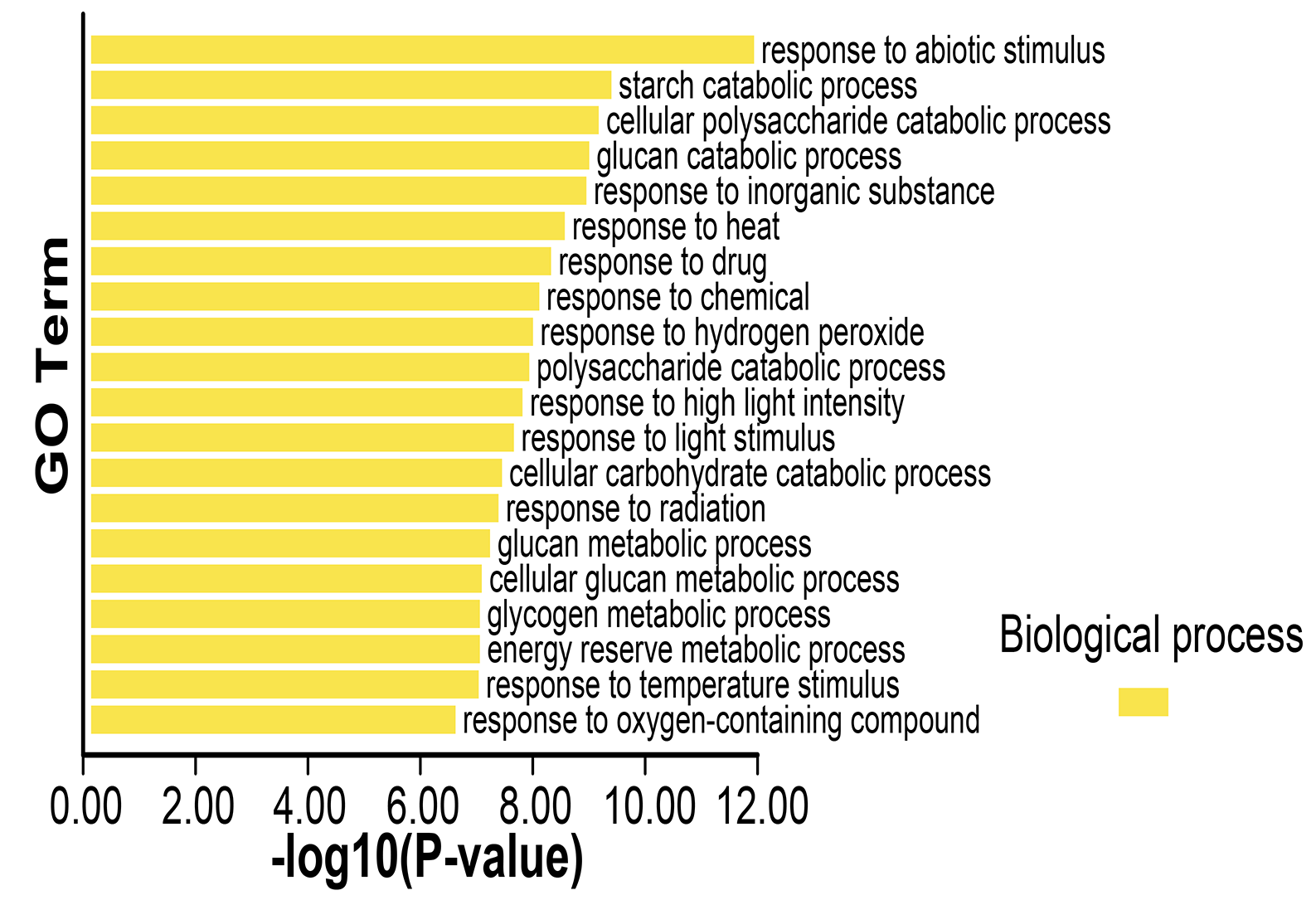

Supplement: Supplementary Figure 2 — Gene ontology (GO) enrichment analysis. According to the adjusted p-value, the top 20 GO terms were shown. [file Image_2.TIF]

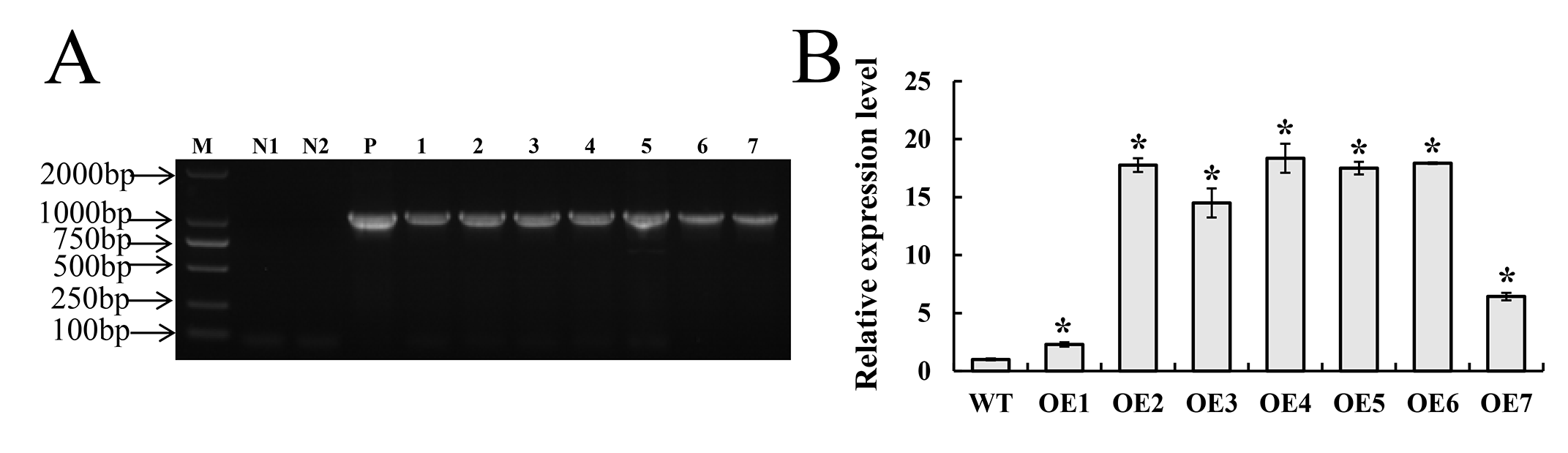

Supplement: Supplementary Figure 3 — Identification of BpLEA14 transgenic lines. (A) The PCR identification of overexpression transgenic plants of BpMYB14, M: DL2000 DNA Marker, N1: negative control (ddH2O), N2: negative control (wild-type of Betula platyphylla), P: positive plasmid, 1-7: different transgenic lines. (B) The qRT-PCR identification of BpLEA14 overexpression (BpLEA14-OE) transgenic plants. Asterisks indicate significant differences by t-test, p < 0.05. [file Image_3.TIF]
