# Supplementary material for: A R2R3-MYB Transcription Factor Gene, BpMYB123, Regulates BpLEA14 to Improve Drought Tolerance in Betula platyphylla
Source: Front Plant Sci. 2021 Dec 10;12:791390. doi: 10.3389/fpls.2021.791390 (PMC8702527; doi:10.3389/fpls.2021.791390)
Supplement: Supplementary file 4 [file Table_1.DOCX]

The primer sequences used in construction for pROK2 and pBI121 plant overexpression vector

| Name | Sequence(5’-3’) |
| --- | --- |
| pROK2-BpMYB123-F | ATCGGATCCATGGGAAGGAGGCCATGCTGTG |
| pROK2-BpMYB123-R | CGGGGTACCTCAACTAATCCATTCGTCCTCTG |
| pROK2-F | GTTGAAGATGCCTCTGCCGACAGTG |
| pROK2-R | CCATTCAGGCTGCGCAACTGTTG |
| pBI121-BpMYB123-F | TGCTCTAGAATGGGAAGGAGGCCATGCTGTG |
| pBI121-BpMYB123-R | ACGCGTCGACCACTAATCCATTCGTCCTCTGAAT |

The primer sequences used in construction for pROK2-RNAi plant repression vector

| Name | Sequence(5’-3’) |
| --- | --- |
| BpMYB123-Cis-F | GCTCTAGAACAAAGGTTGTCATACCATGGC |
| BpMYB123-Cis-R | CGGGATCCGTCCATCCACCACAATCTCACAT |
| BpMYB123-Anti-F | GGACTAGTGTCCATCCACCACAATCTCACAT |
| BpMYB123-Anti-R | CGAGCTCACAAAGGTTGTCATACCATGGC |

The primer sequence of qRT-PCR for *BpMYB123* gene

| Name | Sequence(5’-3’) |
| --- | --- |
| BpMYB123-G-F | GAATCTCAGCCGGTGATTCG |
| BpMYB123-G-R | GTCTCCCTGCTGTGCTGAAC |
| tubulin-F | TCAACCGCCTTGTCTCTCAGG |
| tubulin-R | TGGCTCGAATGCACTGTTGG |
| UBQ-F | TCTGACAGGGAAGACCATA |
| UBQ-R | TCAATTAGAGCTGACCACC |

The primer sequences used to identify candidate *cis*-element sequences.

| Name | Sequence(5’-3’) |
| --- | --- |
| pHIS2-F | GCCTTCGTTTATCTTGCCTGCTC |
| pHIS2-R | CGATCGGTGCGGGCCTCTTC |

The primer sequence of pGADT7-AD and pAbAi vector

| Name | Sequence(5’-3’) |
| --- | --- |
| BpMYB123-AD-F | CGGAATTCATGGGAAGGAGGCCATGCTGTG |
| BpMYB123-AD-R | CGCGGATCCACTAATCCATTCGTCCTCTGAATTTAG |
| T7-F | TAATACGACTCACTATAGGGC |
| AD-R | AGATGGTGCACGATGCACAG |
| pAbAi-BpLEA14-F | CCCAAGCTTGATCCCTGTTGTAGTGTAAGGTTGTG |
| pAbAi-BpLEA14-R | GGGGTACCGAAATTCTTGGCCTTGCTCATCAACTG |
| pAbAi-F | GTTCCTTATATGTAGCTTTCGACAT |
| pAbAi-R | GGTGCGGGATTAGTAATGAACACAAAC |

The primer sequence used for ChIP-PCR

| Name | Sequence(5’-3’) |
| --- | --- |
| BpLEA14-ChIP-F1 | CAGCAGGTTGTCAGGTTATC |
| BpLEA14-ChIP-R1 | CCTGGCTTCAAATTGAAGC |
| BpLEA14-ChIP-F2 | CTCCATCCATATTGCATGC |
| BpLEA14-ChIP-R2 | CAATGCCTTAGGCAGTTAG |
| BpLEA14-ChIP-F3 | GTTTACCATATTAAATGATTAATC |
| BpLEA14-ChIP-R3 | GCATGCAATATGGATGGAG |
| BpLEA14-ChIP-F4 | GTGGCCAAACTACCATCG |
| BpLEA14-ChIP-R4 | GATTAATCATTTAATATGGTAAAC |
| BpLEA14-ChIP-F5 | GTTGTCACTTGAGGTGGTTTAGC |
| BpLEA14-ChIP-R5 | CCCAACCCGCACTCAAACC |
| BpLEA14-ChIP-F6 | GATCCCTGTTGTAGTGTAAG |
| BpLEA14-ChIP-R6 | CTCCCAATGGCCATGATAG |

Construction of primer sequence of pROK2-*BpLEA14* vector

| Name | Sequence(5’-3’) |
| --- | --- |
| pROK2-BpLEA14-F | CGGGATCCATGGCGCAGTTGATGAGCAAGGC |
| pROK2-BpLEA14-R | GGGGTACCTTAAAAGATGTCAGAGACGGTGGGT |
