# Supplementary material for: A R2R3-MYB Transcription Factor Gene, BpMYB123, Regulates BpLEA14 to Improve Drought Tolerance in Betula platyphylla
Source: Front Plant Sci. 2021 Dec 10;12:791390. doi: 10.3389/fpls.2021.791390 (PMC8702527; doi:10.3389/fpls.2021.791390)
Supplement: Supplementary file 7 [file Data_Sheet_1.PDF]

The gene sequence of *BpMYB123* and *BpLEA14*

The gene sequence of *BpMYB123*

ATGGGAAGGAGGCCATGCTGTGCCAAAGAAGGTCTAAACAGGGGTGCATGGTCGGCGCG
TGAAGATAAAATCCTTACCAACTACATTAAAATCCACGGCGAAGGCAAATGGAGAGACCT
CCCTCTAAGAGCTGGGCTGAAGAGATGCGGAAAGAGTTGCCGGCTCCGGTGGTTGAATTA
TCTACGGCCAGATATCAAGAGAGGAAACATTTCTATGGAAGAAGAAGAGCTCATTATCAGA
CTACATAAGCTCCTTGGTAACAGATGGTCACTCATCGCGGGAAGGTTACCGGGGAGAACA
GACAATGAAATCAAGAATTACTGGAACACGAATTTAAGCAAGAGGGTGCACGGTGACAAG
ATTGATGATTTTAATAAACTATATTGTAGTAAATTAGAAAGGAAGGGAAGTATGAGAAACAT
GACTTTGGAGTCCAACCCTGAATCTCAGCCGGTGATTCGGACAAAAGCAGTAAGGTGCAC
AAAGGTTGTCATACCATGGCAAGTAGACAATCAAATGGTGAATAAAAATATAGTTCCGGTA
TGGGATTGTGACACCCCCTCCAGTTCAGCACAGCAGGGAGACAACAATTCGTGCGGTTTT
CTCAAGGATTTTGATATTAATGATCTTTTGATATCGGGAGTGCTGTACTCGGATGATCAAGA
GAAAGAGATGGATGAATGTGAGATTGTGGTGGATGGACAAGACCAGGCCAAGAACATGTC
CGGCTCATGTCCGGAATTGGATTTTCCAGAAGGCAAGATTGAGGAGTCAATGGAGGATGA
AGCAATTGGGGCGGAGAATTGGAGGGGTAGTGATCAGGAGTACCCTTTTCAACCAAATGA
TAATGATCATGCTTTGGATCTCACGACATTGGCATCTTTTCTAAATTCAGAGGACGAATGGA
TTAGTTGA

The gene sequence of *BpLEA14*

ATGGCGCAGTTGATGAGCAAGGCCAAGAATTTCGTGGCAGAGAAGTTGGTCAACGTACAG
AAGCCAGAGGCCTCCATCGATAATGTTGATTTCAAGCGCCTGAGCAGTGACTCGGCGGAG
TACCTCGCTAAGATGTCCGTGAAGAACCCCTACGGACACACCGTGCCCATTTGCGAGATCT
CTTACACCCTCAAGAGTGCTGGCAGGGTGATTGCATCGGGGAAGATGCCGGACCCAGGGT
CACTGAGGGCTAGCGAGACGACAATGCTGGAGTTACCCGTGAAGGTGCCACACAGTGTAT
TGGTGAGCCTGGCAAAGGACATCGGTGCAGATTGCGACATAGACTATGAGCTGGAAGTGG
GTCTCACCGTTGATCTACCCATCATTGGAAACTTTACCATTCCACTCTCCACCAAGGGAGA
GATCAAGCTACCCACCGTCTCTGACATCTTTTAA
